# Lords-and-Ladies (*Arum*) as Food in Eurasia: A Review

**DOI:** 10.3390/plants14040577

**Published:** 2025-02-13

**Authors:** Łukasz Łuczaj, Gizem Emre

**Affiliations:** 1 Faculty of Biology and Nature Conservation, University of Rzeszów, ul. Zelwerowicza 4/451, Building D9, 35-601 Rzeszów, Poland; 2 Department of Pharmaceutical Botany, Faculty of Pharmacy, University of Marmara, 34854 Istanbul, Turkey; gizem.bulut@marmara.edu.tr

**Keywords:** wild edible plants, detoxification, wild food plants, culinary ethnobotany, ethnogastronomy

## Abstract

(1) Background. Although Arum spp. are toxic in their raw state, they are sometimes used as food within their native ranges. (2) Methods. We review the available literature in order to provide an overview of its use and detoxification procedures worldwide. (3) Results. The food use of lords-and-ladies was already mentioned by Theophrastus, Dioscorides, Matthioli, Durante, Gerard, and Sirennius. In the references concerning 19th–21st-century use, seven species were identified: *A. cyrenaicum, A. discoridis, A. italicum, A. maculatum, A. orientale, A. palaestinum*, and *A. rupicola*. Past or current culinary use of the plant has been recorded in Morocco, Libya, the United Kingdom, the Scilly Islands, Germany, Switzerland, Italy, Romania, Ukraine (including Crimea), Czechia, Slovenia, Croatia, Bosnia-Herzegovina, Albania, Georgia, Türkiye, Syria, Palestine, Lebanon, Israel, Iraq, and Iran. (4) In Europe, rhizomes were used, mainly as a famine food. In SW Asia, the aerial parts remain an important element of local cuisine. Several detoxification procedures are used before consumption, such as prolonged boiling, often involving straining the boiled water and lowering the pH with lemon juice, sumac, citric acid, sorrel leaves, or pomegranate juice. (5) Conclusions. Further studies are needed to assess the safety of *Arum* use and record traditional local recipes in SW Asia.

## 1. Introduction

Modern use of vegetables and staples involves using raw, shortly cooked, or fried ingredients. However, in the past, humans also resorted to more complicated procedures of processing plant foods to make them edible by removing toxins and antinutrient agents [1,2,3]. Such procedures involved, for example, pit-cooking overnight, soaking for several days in streams, cooking with ashes, etc., and provided humans with a considerable advantage over other animals. In their Cooking Hypothesis, Wrangham et al. [4] presume that the ability to use fire was the main cause for the reduction in our teeth and bowels, as compared to apes.

The presence of oxalates is often an obstacle to the use of some plants as food and is considered to increase the likelihood of kidney stones [5]. A larger problem arises when oxalates come to form large crystals, which can irritate or even wound our digestive tract. Calcium oxalate crystals have been reported in at least 215 plant families [6]. The presence of crystals in the vegetative tissues of Araceae has been thoroughly reviewed [7]. One genus to have such properties is *Arum*. The shape and size of oxalate crystals may vary between *Arum* species [8]. Although this plant is seen as toxic and indelible in most countries, it is consumed, sometimes frequently, in others.

**Figure 1:**
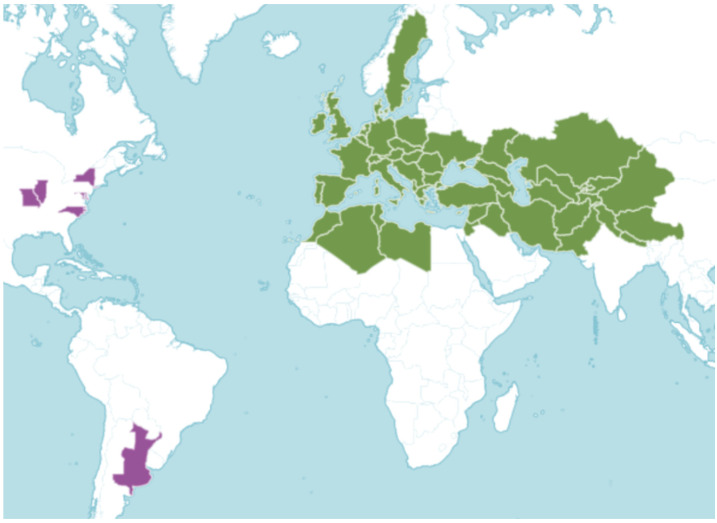
Distribution of the genus *Arum* in the world. Green—native range, purple—non-native range [9].

*Arum* is a genus of plants containing 27 species (Figure 1), which occur in their native range in Europe, Southwest Asia, and the northernmost parts of Africa [9]: *Arum apulum* (Carano) P.C.Boyce, *A. besserianum* Schott, *A. concinnatum* Schott, *A. creticum* Boiss. & Heldr., *A. cylindraceum* Gasp., *A. cyrenaicum* Hruby, *A. dioscoridis* Sm., *A. euxinum* R.R.Mill, *A. gratum* Schott, *A. hainesii* Riedl, *A. hygrophilum* Boiss., *A. idaeum* Coustur. & Gand., *A. italicum* Mill., *A. jacquemontii* Blume, *A. korolkowii* Regel, *A. lucanum* Cavara & Grande, *A. maculatum* L., *A. megobrebi* Lobin, M.Neumann, Bogner & P.C.Boyce, *A. meryemianum* Yıldırım, *A. nigrum* Schott, *A. orientale* M.Bieb., *A. palaestinum* Boiss., *A. pictum* L.f., *A. purpureospathum* P.C.Boyce, *A. rupicola* Boiss., *A. sintenisii* (Engl.) P.C.Boyce, *A.* × *sooi* Terpó, and *A. taiwanianum* S.S.Ying. The greatest richness of species can be found in south-western Asia [9].

A review of the medicinal potential of *Arum* has been conducted by Azab [10]. Also, Ergene and Karaaslan [11] recently reviewed the medicinal properties of one species, *A. maculatum*. However, the culinary ethnobotany of the genus has never been reviewed in full. Unfortunately, all parts of the raw plants from this genus are toxic due to the presence of crystallic oxalates, alkaloids, saponins, toxic odorants, and cyanogenic compounds [10]. Despite this, both the aerial part (mainly leaves) and the tuberous rhizomes of the plant have been widely used as food. Thus, the aim of our article was to review the information on the use of *Arum* as a human food, the parts used, and the culinary techniques applied, with special reference to the detoxification procedure applied.

## 2. Results

### 2.1. Older Historical Accounts

The edibility of arum was first described by Theophrastus in his “Enquiry into Plants,” in the 2nd century BC: “The root of cuckoo-pint (*Arum*) is also edible, and so are the leaves, if they are first boiled down in vinegar” (Book 7, Chapter 12, Paragraph 2) [12].

*Arum* is most likely mentioned in Dioscorides’ De Materia Medica in the chapter “Aron” [13]. Here, its culinary use is clearly mentioned: “Aron sends out leaves similar to those of dracontium, yet smaller and less spotted; a faint purple stalk twenty centimetres long in the shape of a pestle, in which is fruit inclining to a saffron colour. The root—white like that of dracontium—is also [a vegetable] eaten boiled, and is somewhat less sharp. The leaves are preserved in salt for eating. Dried, they are boiled and eaten by themselves. The roots, seeds and leaves have the same strength as dracontium. Particularly the root, applied with bullock’s dung to those troubled with gout, does them good. The root is stored in the same way as the root of dracunculum. In brief it is edible because it is not oversharp. It is also called lupha; among the Syrians it is called alimon, some call it thymon, some, dracontium, and the Cyprians call it colocassion.” (2–197. ARON, 360–361) [13]. Dioscorides also mentions two similar Araceae plants as being the larger and lesser drakontion. One of them is most likely *Dracunculus vulgaris* Schott (which used to belong to the genus *Arum*); the other cannot be clearly identified. Although probably not *Arum*, it cannot be completely excluded that it was some other Araceae species. This smaller drakontion was eaten by the inhabitants of the Balearic Islands in the form of cakes. This information was later attributed to *Arum maculatum* by Pickering [14], followed by Sturtevant [15], although only as a presumption. In our opinion, this information does not apply to *Arum* sensu stricto.

The 11th-century Mishnaic commentator Nathan ben Abraham [16] characterizes the cultivation of *Arum* in the Levant: “‘If arum is covered up with earth in the Seventh Year’ (Sheviit 5:2). This arum that is being covered up with earth does not belong to the [prohibition of] Seventh Year produce, but is rather from last year’s produce. Its manner is such that when it smells the smell of moisture it sprouts. Therefore, they would bury great quantities [of this plant] together and cover them up with dry earth, and the members of one’s household would transfer its leaves to [a place] beneath a roof, so that they will not sprout in the Seventh Year. ‘When arum has remained after the Seventh Year’ (Sheviit 5:4). This arum is the kind that is called qalqās (Taro), being similar to [the leaves of] ar-rakaf (*Cyclamen*), but this is better than it, and its leaves are eaten immediately after sprouting, and it grows quickly, but its roots which are the [plant’s] main fruit does not finish [its growth] and is suitable [for replanting] even after three years [from the time that it is uprooted and buried in dry soil]” (translation from Hebrew [17]).

Both the acridity of *Arum* and its rhizomes’ richness in starch have been reported in early printed herbals in Europe. For example, in Matthioli’s commentaries to Dioscorides [18], translated into English [19]: “Lords-and-ladies, called the lupha by the Syrians, has leaves like those of the dragon arum, but longer and less speckled, and has a reddish stem a palm long, like a pestle, from which the seed, which has the colour of saffron, grows. It has a white root like the dragon arum, but with a less strong flavour, and so it is used in cooking, as are its leaves, seasoned with salt. The dry leaves are also eaten, cooked by themselves. Its root, its seeds and its leaves have the same properties as those of the dragon arum. The root, applied as a poultice together with oxen dung, is particularly good for gout. The root is preserved like that of dragon arum and, since it has a less strong taste, is more often used in cooking.” In his herbal, Durante (1585) reported that, apart from its therapeutic qualities, marvelous flour can be obtained from *Arum* roots [20]. The English herbal of Gerarde from 1597 also mentions the genus [21]: “The most pure and white starch is made of the roots of Cuckoo-Pint; but most hurtful to the hands of the laundress that hath the handling of it for it choppeth, blistereth, and maketh the hands rough and rugged, and withal smarting.” (Chapter 305. Of Cuckoo-Pint, or Wake-Robin). The Polish herbal of Szymon Syreński (Sirennius) mentions many medicinal uses of *Arum* and explains that it can be used as food after boiling two or three times [22] (pp. 637–638).

There are a few interesting 18th-century sources concerning *Arum* use as food. In 1766, the agricultural scholar Giovanni Targioni Tozzetti listed and characterized the wild and cultivated plant species that could be used in times of famine for the Grand Duchy of Tuscany. One of the taxa mentioned was *Arum* (“aro”), which was advised to be used only in emergencies due to its acrid taste. He advised applying one of two proposed procedures for detoxifying edible rhizomes and roots: “Dry tubers and rhizomes, reduce them to flour and then boil the powder in plenty of water for an hour, stirring it with a wooden spoon, until it takes on the consistency of a liquid porridge or polenta. Remove the pot from the fire and let the contents decant for two or three days, then remove the liquid part and collect the flour deposited on the bottom. This flour, once dried, can be mixed with other flours in order to make bread” [23]. In 1781, the French scholar Antoine-Augustin Parmentier published a treatise on edible starchy plants, where he suggested prolonged soaking of grated *Arum* roots (“pied-de-veau,” “*Arum vulgare maculatum*” and “non maculatum,” which probably refers to *A. maculatum* and *A. italicum*) [24]. The leaves of an *Arum* species were eaten by Greeks in Crimea (now the territory of Ukraine occupied by Russia), along with other wild vegetable species, as reported by the traveler Peter Simon Pallas from his 1793–94 journey to this area [25]. He provided the name *Arum maculatum*, although it is more likely that *Arum orientale* was used.

### 2.2. Contemporary or Recent Historical Traditional Use

In the references concerning 19th–21st-century use, seven species were identified [26,27,28,29,30,31,32,33,34,35,36,37,38,39,40,41,42,43,44,45,46,47,48,49,50,51,52,53,54,55,56,57,58,59,60,61,62,63,64,65,66,67,68,69,70,71,72,73,74,75,76]: *A. cyrenaicum, A. discoridis, A. italicum, A. maculatum, A. orientale, A. palaestinum,* and *A. rupicola,* with *A. maculatum* and *A. italicum* are used most widely, probably due to their large geographical ranges (for details see Section 2.3 and Table 1).

There is a clear division between the uses of *Arum* for food in Europe and Southwest Asia. In Europe, the plant is generally considered toxic, and the last cases of using lords-and-ladies as food come from times of severe food scarcity in the Balkans (e.g., in Croatia and Bosnia-Herzegovina) during World War II. Earlier, it was used in the present territory of the United Kingdom, Scilly Islands, Germany, Switzerland, Romania, Ukraine, Italy, Czechia, Slovenia, Coratia, Bosnia-Herzegovina, and Albania (for details see Section 2.3). The only current use of *Arum* reported from Europe comes from Middle Eastern refugees in Germany, who collect *Arum maculatum*, native to Germany, probably as a substitute for their native *Arum* species [51], although it is not impossible that they used the same species in their homeland.

In the Caucasus, the use of *Arum* leaves to make phkhali vegetable balls has been reported by several field studies in Georgia, but the practice seems to be dying out. In Southwest Asia (Türkiye, Palestine, Lebanon, Israel, Syria, Iraq, Iran), arums are still used locally and form an important part of the traditional cuisine. Leaves are added to soups, stews, and salads or even used as sarma wrapping. A variety of detoxification techniques are employed, especially boiling combined with discarding the water and adding other sour plants; e.g., sumac (*Rhus coriaria* L.) fruits, sorrel (*Rumex* sp.) leaves, pomegranate (*Punica granatum* L.), or lemon (*Citrus × limon* (L.) Osbeck) juice (Table 2). There are only two notes on the food use of *Arum* from Northern Africa, probably due to the rarity of the genus there.

### 2.3. List of Species of Arum Used as Food with Relevant References

Information is given in the following order: country (in bold), local name (underlined italics), information on use, and source.

***Arum cyrenaicum*** Hruby.

**Libya.** The rhizomes are used as food [27].

***Arum dioscoridis*** Sm.

**Türkiye.** *Ağı, Ağı otu, Ağu, Andırın doktoru, Asalan, Elkabartan, Gavur pancarı, Kari, Kardi. Kabarcık, Kabargan, Kına otu, Kınaağısı, Kınaağusu, Papuçağusu, Tirşik*, *Tirsin, Yılan ağısı, Yılanbıçağı, Yilanekmegi, Yılan burçağı, Yılan pancarı, Yılan yastığı, Zilke araba.* The leaves are boiled in water and then cooked as sarma wrapping [28]. Alternatively, the leaves are boiled in water, strained, and boiled again for potherb, called “tirşik” [28]. The leaves are used for sarma wrapping in south and southeastern Anatolia [29]. After prolonged boiling with lemon or citric acid, the leaves are used as sarma wrapping, or eaten as a vegetable with yogurt and salt in Karaisali near Adana [30]. The leaves are boiled once in water, drained, boiled a second time, and then cooked for potherb [28,31,32]. In Karaköprü (Şanlıurfa), the leaves are cooked in a soup called “kardi.” The leaves are also dried for further use [33]. In Mersin, the leaves are boiled for food [34].

***Arum italicum*** Mill. 

**Scilly Islands.** This plant was in cultivation for seven years in Guernsey for the purpose of making arrowroot from its corms [15]. **Italy.** *Aro, Gigaro, Pan de Serpe.* The plant is used to make starch for bread in times of food scarcity in many parts of Italy; e.g., Veneto, Tuscany, Liguria, Puglia, and Basilicata [23]. In 1918, Pantanelli published a guidebook on how to process *Arum* for starch [35]. **Slovenia.** *Štrkat, Zminac, Zmijinac*. Rhizomes cooked [36]. **Croatia.** *Gujino zelje, Kozlac, Štarkavac, Strtok, Zminac, Žuminac*. The leaves, or more rarely rhizomes (e.g., on Cres), were boiled as a famine food during World War II and before, on at least seven islands [37], including Krk, and in the Poljica region [38]. The rhizomes are widely used in coastal Croatia as an ingredient of emergency bread during World War II [39]. The leaves are used in wild vegetable mixes in Dalmatian Zagora [40]. **Bosnia-Herzegovina.** *Kazalac, konjska blitva, kozlac*. The rhizomes are used for mush and bread [41]. The rhizomes are used as a famine food (boiled or roasted but still pungent when eaten) in southern Herzegovina [42]. **Albania.** *Kelkass*, As *Arum byzantinum*; pungent roots were eaten in the Shkodra area in the 19th century [43,44,45]. **Türkiye.** *Arko lahanası, Ayı lahanası, Çiçek otu, Domuzyandıran, Gabarcık, Pezük, Tirşik, Yılanbıçağı Yandıran, Yılan otu, Yılansoğanı, Yılanyastığı, Yılanzehiri, Zehirotu*. The leaves are boiled in water, which is later discarded and then cooked (like spinach) [28]. The leaves are cooked with wheat, yogurt, flour, and chickpeas, and soured and cooked as a soup [27]. The leaves are also used in soup after prolonged cooking (Izmit) [46]. **Georgia.** *Kala k’oda, Qalakoda.* The leaves are used to make phkhali [47], especially after prior drying [26], reported also as ***Arum albispathum*** Stev. [26]. **raq** (Northern Iraqi Kurdistan). *Kari, Kardu, Kardun, Karduw, Kordi, Kori, Nuta*. After treatment with sumac and straining, the leaves are used either as a wrap in the preparation of dolma or chopped and mixed with eggs for omelets [48]. As *Arum italicum* Mill. subsp. *albispathum* (Steven ex Ledeb.). The leaves are boiled and then macerated in “sumac water” (suspension of water and sumac fruits) or water and lemon juice, sometimes fried, then consumed with the possible addition of tahini or tomato sauce; lacto-fermented [49]. 

***Arum maculatum*** L.

**England.** *Adam-and-Eve, Bobbins, Cuckoo Pint, Lord-and-Ladies, Starch-Root, Wake Robin.* “The thick and tuberous root, while fresh, is extremely acrid, but by heat its injurious qualities are destroyed, and in the isle of Portland the plant was extensively used in the preparation of an arrow-root.” [15]. “Arum arrowroot is procured from the tubers of *Arum maculatum*, the common ‘cuckoo pint’, ‘wake robin’ and ‘lords and ladies’: it is prepared chiefly in Portland island; hence it is generally called ‘Portland arrowroot’. The mode of preparation is very similar to that adopted with other arrowroots; the tubers are pounded in a mortar, the pulp repeatedly washed, and water is subsequently strained. As the tubers are very acrid, great care is required in the washing and straining, so that the acridity may be completely removed. (...) The starch granules of *Arum* arrowroot are very small, and, except in size, they resemble those of Tacca arrowroot; but this difference is sufficiently constant and considerable to ensure the ready identification of the two kinds.” [50]. **Italy.** *Aro, Gigaro, Pan de Serpe.* The plant is used to make starch for bread in times of food scarcity in many parts of Italy; e.g., Veneto, Tuscany, Liguria, Puglia, and Basilicata [23]. In 1918, Pantanelli published a guidebook on how to process *Arum* for starch [35]. **Switzerland.** *A. maculatum* leaves were used in some dishes, mainly in a medicinal context [76,77,78,79]; e.g., for cough sufferers, a few finely chopped leaves of the herb would be baked into an omelet, while half a cup of tea made from the herb was drunk in the morning and evening with a little sugar and old white wine [77]. The aerial parts of *Arum* are baked into “Arontotsch,” a cake given to children to treat “corrupt blood” [78]. These medicinal practices may be the vestige of an even older food use. **Germany**. *Aronstab* and numerous other names were reported by Marzell [76]. In the past, the plant’s leaves were occasionally added in very small amounts to the so-called Nine Herb Soup eaten on Maundy Thursday before Easter [76]. The root was eaten as a stomachic [76]. *Arum maculatum* is a protected species that does not normally require special protection due to its poisonous components. However, employees of the Lower Nature Conservation Authority (UNB) and the environmental advisory office of the Plön district were all the more astonished to find the plant being picked by the sack on Plön’s Princes’ Island by immigrants from the Middle East. In two cases, plucked leaves and flowers with a total weight of almost 100 kg were seized. The immigrants stated that they would cook the greens for a long time and then eat them like spinach [51]. **Czechia.*** Blázivec.* The rhizomes, powdered to flour, are used as famine food [52]. **Slovenia**. *Kačjak, Kačje zelje, Kačnik, Kozlac.* The rhizomes are cooked [36]. **Croatia**. In Slavonia (region of Croatia), rhizomes were made into a kind of bread [15]. **Bosnia-Herzegovina**. *Kozlac.* The rhizomes are used for mush and bread (probably as a famine food) [41]. **Albania.** The rhizomes were cooked and eaten in Albania [15]. **Ukraine.** The rhizomes were eaten by Hutsuls in the Carpathians after boiling, leaching, and drying [1]. **Türkiye.** *Mayasıl otu, Pancar, Tirşik, Yılan burçağı, Yılan otu, Yılanekmeği, Yılanyastığı*. The plant is used as sarma wrapping in Western and Central Anatolia [53]. The plant is used to make tirşik soup in Adana, Osmaniye, and Kahramanmaraş provinces in the eastern Mediterranean region of Turkey [54,55]; for the soup recipe, see Appendix A. **Syria.** *Louf.* Soup is made with *Rumex acetosa* L, *Rhus coriaria*, bulgur (cracked, parboiled groats of *Triticum durum* Desf.), and olive oil [56] (see Appendix A). The green parts have to be boiled for 2–3 h and the water discarded; then the abovementioned ingredients are added and the soup is boiled again (https://www.youtube.com/watch?v=ypVr1gmdqmc&t=194s, accessed date 12 February 2025) **Iran.** *Kardu, Khaz.* The aerial parts of the plant are used in soups and stews made by Sawramani Kurds [57]. 

***Arum orientale*** M.Bieb.

**Türkiye***. El kabartan, Gavurotu, Kabarcık, Kabarağı, Kardun, Kardu, Kardı, Yılanbıçağı, Yılancücüğü, Yılanyastığı.* The leaves are eaten as a “salad,” with no preparation method specified [58]. Also as ***Arum elongatum*** Steven. Fresh rhizomes are eaten after boiling and removing water, and dried leaves are cooked [59]. **Georgia.** *Kala k’oda, Kalakoda.* The leaves are used to make phkhali after prior drying [26]. The leaves are used to make phkhali [47]. 

***Arum palaestinum*** Boiss.

**Palestine**. *Lufe*. The leaves are cut and boiled, the water is decanted several times to remove toxic substances, and then the leaf parts are fried in olive oil and garnished with lemon; they are also used as herbal tea [60]. The plant is one of the main wild edible plants in Palestine [61]. **Lebanon.** *Louf Falastini*, *Louf.* Stew is made with previously boiled and strained leaves [62]. **Israel.** *Luf.* The plant is mentioned several times in the Mishnah and its commentaries as well as in the Talmud, most likely *A. palaestinum* [16,17,63]. Lemon juice or sorrel (*Rumex* sp.) is used as a detoxifying agent for the plant’s edible leaves [64] (see Appendix A and [65]).

***Arum rupicola* Boiss.**

**Türkiye.** *Garibent, Gavur pancarı, İlhan burçağı, İlhanbıçağı, Kahri, Nıviç, Yılan burçağı, Yılan otu, Yılanbıçağı, Yılandili, Yılanekmeği, Yılanyastığı*. Leaves are boiled in an acidic environment with sumac, “cheese water” (probably whey), pomegranate juice or citric acid, and strained and fried with onion in Yeşilli (Mardin) [66]. Leaves used for food after drying, Ağrı province [67]. Rhizomes used for rennet production or boiled in bulgur pilaf or soup (Hizan, Bitlis) [68]. As ***A. dentruncatum*** var. ***dentruncatum.*** Leaves are boiled, then cooked again after straining [28]. As ***A. elongatum*** var. ***detruncatum*** H.Riedl. Leaves are used for sarma: “The collected fresh leaves are boiled until they become soft and the filling (onion, tomato paste, black pepper, parsley and rice) is added and wrapped. After cooking, it is consumed with yoghurt.” (Mihalıççık district, Eskişehir) [69]. As ***A. conophalloides***. Aerial parts cooked as a stew or rice vegetable dish in Çatak (Van) [70] and in Geçitli (Hakkari) [71]. As ***A. conophalloides****;* used as sarma wapping or eaten as a vegetable with yoghurt and salt, both after prolonged boiling with lemon or citric acid, in Karaisali [29]. **Iraq.** *Kari, Kardu, Kardû, Kardun, Kordi, Kori, Nuta, Xaz*. Leaves cooked with dried mulberries, ground wheat and pomegranate sauce (and possibly sumac); as wrapping leaves for dolma [72]. Frequently used by Kurds and Yazidis in central Iraqi Kurdistan: boiled and then macerated in “sumac water” (suspension of water and sumac fruits) or water and lemon juice, sometimes fried, then consumed with the possible addition of tahini or tomato sauce; lacto-fermented [49]. **Iran.** *Kardim.* As ***A. conophalloides****,* leaves used in medicinal soups [73]. 

***Arum*** **sp.**

**Morocco.** *Irni, yerni*. The rhizomes were used as famine food [74]. **Romania and/or Moldova**. The rhizomes were used in Wallachian and Moldavian cuisine in the 19th century [1]. **Türkiye.** *Acı manca, Ağı, Ağu, Ağu kınası, Ayı marlı, Basır otu, Bilakama, Dağ sorsalı, Domuz lahanası, Domuz pancarı, Filkulağı, Garia, Kabarcık, Kardi, Kardon, Kari, Kariya belik, Livik, Nifik, Tirşik otu, Vilinç, Yaldıran, Yılan ağusu, Yılan burçağı, Yılan üzümü, Yılan yarpuzu, Yılanbıçağı, Yılancık, Yılandili, Yılan yastığı, Yiviş*. Tuzlacı [28] describes several methods of using unidentified *Arum* leaves in consumption without specifying the species: 1. They are boiled in water, strained, boiled again, and then cooked with other ingredients (like spinach). 2. They are finely chopped and mixed thoroughly with yogurt and salt in hot water. Then bulgur or flour is added and cooked. After waiting for 10–12 h, the food starts fermenting and is cooked again. 3. They are boiled in water with the addition of yogurt. 4. The leaves are boiled in water and cooked with bulgur wheat or rice. 5. The leaves are crushed with *Rhus coriaria*, then boiled. 6. The leaves are boiled for sarma. 7. Dried leaves are cooked with onion. 

## 3. Discussion

The use of *Arum* rhizomes seems to be mainly obsolete. In the past, severe food shortages likely caused people to seek starch-rich foods such as rhizomes and bulbs [1,2,3,75]. Digging out plant parts is a more destructive method [80,81] of dealing with the plant population, and was used either when the human population density was lower or food shortages were severe.

In our review, we concentrated on food uses; however, the literature on the medicinal uses of *Arum* species is much vaster (e.g., [82,83]) and the genus also needs a review in this aspect. Some of the medicinal uses are indirectly connected with consumption. For example, Marzell [76] describes the use of *A. maculatum* roots as a stomachic in Germany. He reported that in the 16th century, the arum root was already well known as a stomachic, because it was called “German ginger” at the time. The arum root was supposed to make people “happy to eat”, and an old handwritten pharmacopeia from Switzerland even stated how one can “eat and digest a whole sheep” by eating arum root the size of half a hazelnut before going to bed. Furthermore, he mentions information that farmers in Saxony, and especially the beer drinkers, ate a lot of this root, drank a lot afterwards, and rarely needed other medicines.

Apart from its specific medicinal properties, in **Türkiye**, dishes made from *Arum* are imbued with symbolic meaning, as they are generally associated with health. For example, people believe that those who eat “tirşik” (*Arum* soup) once will not get sick that year [28]. The consumption of this soup has many symbolic connotations. As Yayla [54] reports: “It is critical to note that the yoghurt used in the dish is sour. The predominance of a sour taste in the Tirşik dish made with sour yoghurt is an important feature desired by people. There are different practices among people for the souring of the Tirşik dish. As people associate a sour taste with quarrelsomeness, the clothes of the most quarrelsome person in the family are put on the cooking vessel during the fermentation process. In addition, the dish is prepared by dedicating it to the quarrelsome person, orally. In local culture, leavening is defined as an improvement of the dish. To improve the dish, theatrical fights are also held in folk rituals during the preparation of the dish. It is also thought that the more violent the fight, the sourer the dish will be. In Turkish, the condition of not having good interpersonal relationships is defined by the word ‘limoni’ (lemon-like). As the word limoni derives from the word ‘lemon’, it is also associated with sourness. Thereby, the limoni (unsettling) atmosphere created while making Tirşik suggests that the dish will be sourer. For this reason, an unsettling atmosphere is created around the dish. Creating such an unsettling atmosphere requires the presence of several people. This demonstrates that Tirşik is a dish prepared in a ritual with a group of people.”

*Arum* consumption is also a part of a local cuisine’s identity, so it is not surprising, that when migrants from the Middle East (probably Syria) arrived in Germany, they started collecting the leaves of local *Arum maculatum* as this genus is such an important plant for their cuisine and they could not buy it [51].

More research is needed on *Arum* as a food, especially for what concerns its detoxification procedures (Table 2). Consumed without proper processing, the plant causes irritation of the skin and digestive tracts, swelling, and nausea. However, lethal poisoning has not been recently reported in Europe, despite numerous cases of accidental ingestion [84]. On the other hand, in Palestine, where *A. palaestinum* and its use are common, the species causes frequent poisonings among children consuming it raw [85]. Kisslinger’s self-ingestion experiments showed that although the consumption of *Arum maculatum* berries led to long-lasting, unpleasant irritation, it did not cause harm to his health [86]. The presented evidence suggests that a low pH is required for oxalates to be removed from the consumed plant tissue. Usually, sumac fruits are used, but also lemon juice, citric acid, pomegranate juice, or sorrel leaves are applied. Another method is prolonged boiling and then straining, sometimes twice (Table 2).

Recording the local uses of a plant normally considered toxic can be very useful for new movements of “city” foragers [87,88], helping them avoid poisoning. While *Arum maculatum* is relatively abundant in Western Europe, it is easily confused with other wild edibles that do not require detoxification; e.g., sorrel (*Rumex* spp.) or ramsons (*Allium ursinum* L.). Documenting local food traditions helps to preserve local cuisines, as previously examined in studies on tirşik soup, which has become a regional attraction in parts of Türkiye [54,55].

It is also likely that the presented preparation procedures work for the detoxification of the plant, not only by removing oxalates but also by getting rid of other harmful substances, such as saponins or cyanogenic glycosides, that are present in the genus [10,11]. Citrate is a well-known inhibitor of the formation of calcium oxalate [89,90]. We may also assume that organic acids contained in lemon juice, sorrel, pomegranate juice, and sumac berries may form calcium salts, turning calcium oxalate into soluble oxalic acids, which are not irritants. Even sorrel, which is mainly seen as a source of oxalic acid, contains large amounts of citric acid [91]. It is possible that vinegar (with its acetic acid), as proposed by Theophrastus [12], works in a similar way. The traditional cooking techniques used for *Arum* detoxification presented in this review would support the view that the formation of calcium oxalate crystals may be reversible (see also [92]).

With our current knowledge of *Arum*, we are unable to tell the extent to which cooking and detoxification procedures for one species or population can be transferred to other species or geographic areas. A similar situation may occur with tannins in *Quercus* acorns, which vary widely in their tannin content—some can be eaten raw, but most require some degree of leaching [93]. On the other hand, we can presume that these detoxification procedures can be used for most *Arum* species, as the different species within the genus are probably not distinguished by the local population and treated in the same way [28,75]. Nevertheless, this issue requires further study.

## 4. Materials and Methods

We searched the existing literature concerning wild edible plants of Europe and Southwest Asia, within the limits of the geographical range of *Arum*. The search was enhanced by using various combinations of keywords online (e.g., ARUM and FOOD, ARUM and EDIBLE), as well as adding country names (e.g., ARUM and PALESTINE and FOOD). We covered both primary sources and secondary publications from Antiquity up to the present.

## Figures and Tables

**Table 1:** Use of *Arum* species in different countries.

	*A. maculatum*	*A. cyrenaocum*	*Arum* sp.	*A. italicum*	*A. orientale*	*A. dioscoridis*	*A. rupicola*	*A. palaestinum*
Czechia	R							
England	R							
Switzerland	L							
Italy	R			R				
Slovenia	R			R				
Bosnia-Herzegovina				R				
Scilly Islands				R				
Albania				R				
Libya		R						
Romania/Moldova			R					
Croatia				LR				
Ukraine	R							
Crimea (18th century)			L					
Germany	LR							
Georgia				L	L			
Türkiye				L	RL	L	L	
Syria	L							
Iraq				L			L	
Iran	L						L	
Palestine								L
Israel								L
Lebanon								L

L—leaves; R—tuberous rhizomes.

**Table 2:** Detoxification procedures and detoxifying agents used apart from prolonged boiling.

	*A. dioscoridis*	*A. italicum*	*A. maculatum*	*A. orientale*	*A. palaestinum*	*A. rupicola*	*Arum* sp.
Water strained after boiling			[50]	[59]	[62]	[28,30,66]	[23]
Drying	[33]	[26]		[59]			[13,18,23]
Sumac (*Rhus coriaria*)		[48,49]	[56]			[49,67,72]	
Yogurt	[30]					[30,69]	
Lemon (*Citrus* x *limon*) juice	[30]	[49]			[64]		
Citric acid	[30]					[66]	
Pomegranate (*Punica granatum*) juice						[66,72]	
Sorrel (*Rumex* sp.)			[56]		[64]		
Vinegar							[12]
Whey							
Soaking in cold water						[66]	[24]

## Data Availability

All the analyzed data are provided within the text.

## Appendix A

### Appendix A.1. Tirşik Çorbası—A Recipe from Turkey

Ceylan and Sahingoz [55] provide a recipe for soup made from *Arum maculatum* leaves, as paraphrased below.

Ingredients:

500 g cuckoo pint;

250 g yogurt;

75 g dövme, a wheat product frequently used in Turkish cooking—obtained by boiling and drying the hard wheat and then pounding and removing the shell;

75 g chickpeas;

2 l water;

1 tsp. salt;

500 g flour.

The arum leaves should be finely chopped. Then rinse the leaves twice. Heat the water to 50 °C. Mix the whipped yogurt with the leaves and water, and stir. Also, add chickpeas and dövme to the mixture. Level the mix with a spoon. Make a thin layer on the surface of the cuckoo pint with the remaining flour. Close the lid so that the mixture has no contact with air. Keep the temperature at around 27–30 °C and wait for over 12 h. Check if the mix is rising, which is a sign of fermentation. If it is not, wait another 3–4 h. Remove the flour from the top. Bring the mix to the boil, stir occasionally, and continue boiling for about 150 min.

Yayla [54] provides different proportions of ingredients:

1 kg *Arum maculatum* leaves;

4 l water;

250 g yogurt;

175 g flour;

10 g cracked wheat;

10 g chickpeas;

1 tsp. salt.

### Appendix A.2. Louf Soup from Syria

“Louf is a traditional soup consumed only in some parts of the Syrian coastal mountains, especially by the Alawite and Ismaili cultural-religious groups. The soup has a sour-astringent taste with a thick texture. *Arum maculatum* is the main ingredient in Louf soup; its dark-green leaves are loosely cut and steamed for around half an hour. Afterwards, olive oil and bulgur are added, then the water of boiled *Rhus coriaria* is added after disposing of *Rhus coriaria*. *Arum maculatum* contains a high amount of calcium oxalate, which is a toxic compound; however, as local people are aware of this toxicity, it is thus prepared by boiling for more than an hour to detoxify it. The dish is characterized as a winter soup, as *Arum maculatum* is usually available from December to April. Some informants reported that they store it in glass jars in the refrigerator and consume it throughout the year” [56].

### Appendix A.3. Luf (Arum Palaestinum) Preparation—A Recipe from Israel

“With the de-spining finished, Hal’la showed me how to take a pile of the folded leaves, wrap a big leaf around them, then slice the whole roll in half. Then she put one rolled half on top of the other and cut through them to make thin shreds of the leaves. When that was done, we chopped onion, sautéed it in a generous pool of olive oil, and when it was transparent, added the shredded luf. Soon it was emitting plenty of liquid but Hal’la kept stirring the mixture so it would steam away. This, she assured me, would prevent any unpleasant sensation in one’s mouth from eating it. When most of the liquid had dissipated, she added another full cup of water and again stirred constantly until that, too was gone. I squeezed juice from about 5 small lemons and we added that to the mixture—after about an hour of cooking, the luf was a deep green, thick stew, and ready to eat” [65].
